# Small putative NANOG, SOX2, and SSEA-4-positive stem cells resembling very small embryonic-like stem cells in sections of ovarian tissue in patients with ovarian cancer

**DOI:** 10.1186/s13048-016-0221-3

**Published:** 2016-03-03

**Authors:** Irma Virant-Klun, Natasa Kenda-Suster, Spela Smrkolj

**Affiliations:** Department of Obstetrics and Gynaecology, University Medical Centre Ljubljana, Slajmerjeva 3, 1000 Ljubljana, Slovenia

**Keywords:** Human, Ovary, Cancer, Very small embryonic-like stem cells, VSELs, Stemness, Pluripotency, Immunohistochemistry

## Abstract

**Background:**

In previous studies it has been found that in cell cultures of human adult ovaries there is a population of small stem cells with diameters of 2–4 μm, which are present mainly in the ovarian surface epithelium and are comparable to very small embryonic-like stem cells (VSELs) from bone marrow. These cells are not observed by histopathologists in the ovarian tissue due to their small size and unknown clinical significance. Because these cells express a degree of pluripotency, they might be involved in the manifestation of ovarian cancer. Therefore we studied the ovarian tissue sections in women with borderline ovarian cancer and serous ovarian carcinoma to perhaps identify the small putative stem cells in situ.

**Methods:**

In 27 women with borderline ovarian cancer and 20 women with high-grade serous ovarian carcinoma the ovarian tissue sections were stained, per standard practice, with eosin and hematoxylin staining and on NANOG, SSEA-4 and SOX2 markers, related to pluripotency, using immunohistochemistry. We focused on the presence and localization of small putative stem cells with diameters of up to 5 μm and with the nuclei spread over nearly the full cell volume.

**Results:**

In ovarian sections of both borderline ovarian cancer and serous ovarian carcinoma patients we were able to identify the presence of small round cells complying with the above criteria. Some of these small cells were NANOG-positive, were located among epithelial cells in the ovarian surface epithelium and as a single cell or groups of cells/clusters in typical “chambers”, were found only in the presence of ovarian cancer and not in healthy ovaries and are comparable to those in fetal ovaries. We envision that these small cells could be related to NANOG-positive tumor-like structures and oocyte-like cells in similar “chambers” found in sections of cancerous ovaries, which could support their stemness and pluripotency. Further immunohistochemistry revealed a similar population of SSEA-4 and SOX2-positive cells.

**Conclusions:**

We may conclude that putative small stem cells expressing markers, related to pluripotency, are present in the ovarian tissue sections of women with borderline ovarian cancer and high-grade serous ovarian carcinoma thus indicating their potential involvement in ovarian cancer.

**Electronic supplementary material:**

The online version of this article (doi:10.1186/s13048-016-0221-3) contains supplementary material, which is available to authorized users.

## Background

Ovarian cancer is an aggressive disease affecting a large population of women worldwide. It is difficult to diagnose early because of a lack of symptoms, and it can be quite resistant to treatment with radiotherapy and chemotherapy. Also, borderline ovarian cancer represents a difficult task for diagnosis, and it can proceed into an aggressive, resistant, and lethal disease. Ovarian cancer often results in the remission of disease and lethality, and its manifestation is still poorly understood. The aggressive character of ovarian cancer is attributed to cancer stem cells [1–5]. Ovarian cancer stem cells are widely researched using established markers such as aldehyde dehydrogenase (ALDH1) [6–10] or leucine-rich, repeat-containing G protein-coupled receptor 5 (LGR5) [11–15], mostly in ovarian cancer cell lines (e.g., OVCAR3, SKOV3, IGROV1) in vitro. In spite of that, there is still no generally accepted population of ovarian cancer tumor-initiating stem cells in terms of their phenotype and molecular status. The study of ovarian cancer lines does not necessarily reflect the situation in vivo. Moreover, there might be different populations of stem cells co-acting in the manifestation and invasion of ovarian cancer at different stages of progression [16–18].

Our group has discovered small stem cells with diameters of 2–4 μm in adult human ovaries [19, 20] which can be sorted from the ovarian tissue [21] and pushed into developing primitive oocyte-like cells in vitro in the presence of follicular fluid containing several substances important for growth and maturation of oocytes [22]. These small stem cells are mostly located in the ovarian surface epithelium [22, 23] and express several genes related to pluripotency (e.g., *NANOG*, *SALL4*, *LIN28B*, *LEFTY*, *ZIC3* and *LEFTY1*) and germinal lineage (e.g., *VASA*/*DDX4*), especially primordial germ cells (PGCs) (e.g., PRDM14), as evidenced by transcriptomics [24]. They have also been found in adult human ovaries by some other research groups [25] and in the ovaries of other mammalian species such as rabbit, sheep, monkey [25], mouse [26], and pig [27]. Due to the character of these small stem cells, the possibility is not excluded that they could also be involved in the manifestation of ovarian cancer. Ovarian small stem cells are quite comparable to very small embryonic-like stem cells (VSELs) from human bone marrow [28, 29] and peripheral [30] and umbilical cord blood [31], discovered by the Ratajczak research group. The origin of these VSELs has been suggested to lie in the embryonal epiblast and then persist in adult human tissues and organs from the embryonic period of life in a quiescent state [32–35]. VSELs seem to be epigenetically “locked” to prevent teratoma formation in human adult tissues and organs [35] but are proposed to form tumors upon inappropriate conditions in the body [36]. Some other groups reported on the oogonial stem cells in adult human ovaries which may represent the descendants of small ovarian stem cells [37]. Furthermore, in several studies it has been reported that mesenchymal stem cells (MSCs) can also express some markers of pluripotency, are important for spreading and the invasion of tumors, and support cancer stem cells [38–49]. Putative ovarian MSCs have already been successfully cultured and differentiated in vitro in humans [50]. Moreover, the epithelial-mesenchymal transition has been proposed to play an important role in the manifestation of ovarian cancer and its resistance to therapy [51–67].

The aim of this study was to identify potential ovarian stem cells in situ in ovarian sections of women with borderline ovarian cancer or high-grade serous ovarian carcinoma using immunohistochemistry for pluripotency-related NANOG, which is known to be involved in proliferation and self-renewal of pluripotent stem cells [68]. The marker NANOG has been analyzed on account of our previous finding that this marker is strongly expressed in small stem cells from adult human ovaries, [24] and its expression in cancerous ovaries has already been related to ovarian cancer in terms of poorer outcome in ovarian epithelial malignancies [69]. Furthermore, the expression of SOX2 and SSEA-4 markers, related to pluripotency, was analyzed to compare it with NANOG expression in ovarian sections. Our special emphasis has been devoted to small ovarian stem cells, which are usually not monitored by histopathologists because of their small size and unknown clinical significance.

## Methods

This study has been approved by the Slovenian Medical Ethical Committee (Ministry of Health of the Republic of Slovenia, No. 135/09/09 and 154/07/10) in the frame of ovarian stem cell research and is in compliance with the Helsinki Declaration. The ovarian tissue sections of 47 women: 27 women with borderline ovarian cancer and 20 women with high-grade serous ovarian carcinoma were collected at our Histopathology Unit to perform histopathological diagnosis and were then included into this study. Ovarian tissue sections were stained by hematoxylin and eosin (HE) staining, common in daily medical practice, which resulted in blue-stained nuclei of cells. Using immunohistochemistry, the ovarian tissue sections of 19 women with borderline ovarian cancer and 20 women with serous ovarian cancer were analyzed on pluripotency-related marker NANOG, expressed in the nucleus, to identify the potential stem cells expressing this marker and their localization in the ovary. We particularly focused on small putative stem cells with diameters of up to 5 μm expressing a degree of pluripotency which had previously been discovered in ovarian cell cultures in vitro. In three women with high-grade serous ovarian carcinoma the ovarian sections were further stained for pluripotency-related markers SSEA-4 and SOX2.

### Immunohistochemistry (IHC) for NANOG

IHC analysis was performed on a tissue microarray (TMA) of tissue samples from women with ovarian borderline cancer and high-grade serous ovarian carcinoma. After evaluation of all HE stained sections of ovarian tissue/tumor, the representative areas of the ovarian/tumor tissue were marked and biopsy was performed using a 2 mm core needle. The corresponding ovarian/tumor tissue was then transplanted to multitumor paraffin blocks. Consecutively, 3–5 μm sections were cut from each multitumor block. The paraffin sections were then placed on silane-coated slides (Menzel-Glaser Superfrost) and dried in a dryer for one hour at 60 °C. The immunohistochemical staining was performed in an automatic slide stainer (Ventana BenchMark GX). After deparaffinization, antigen retrieval (HIER) was performed at pH 7–8 for 48 min with CC1 Ventana reagent. This was followed by hand application of the primary antibody. To do this, the slides were incubated with rabbit anti-human NANOG monoclonal antibody (ab109250, Abcam Cambridge, MA, USA) at dilution 1:25 for 30 min at 37 °C. For antigen detection, a Ventana OptiView Kit was used. The testicular embryonal carcinoma tissue, treated in the same way, was used as a positive control, and elimination of the primary antibody served as a negative control. In each woman up to 2 ovarian sections were stained. The stained slides were monitored under a light microscope (at magnifications up to 1000x), common in histopathology, but also under an inverted microscope (at magnifications up to 200x) to better elucidate the potential stem cells. After HE staining, the cell nuclei were stained blue and the NANOG-positivity was expressed by the color brown. We focused on nuclear NANOG staining which matched with blue HE staining.

### Immunohistochemistry for SSEA-4 and SOX2

Paraffin embedded ovarian tissue was cut into 4 μm thick sections, mounted on silanized slides and dried at 37 °C overnight. Next morning the sections were deparaffinised with xylene (2 washes for 10 min) and rehydrated through a graded ethanol series; the sections were rinsed twice for 2 min in absolute ethanol and then four times for 1 min subsequently in 96 %, 80 %, 50 % and 30 % ethanol. Then the sections were rinsed for 2 min in tap water. For antigen retrieval the sections were boiled in Target Retrieval Solution (Dako) for 10 min at low power in a microwave oven. After cooling down, the sections were rinsed with PBS. When intracellular antigen was observed, the sections were permeabilized with 0.3 % Triton X-100 for 10 min and rinsed with PBS. The following procedures were the same for detecting intracellular or surface antigens. Unspecific binding sites were blocked with 10 % fetal bovine serum (the sections were incubated for 30 min) and then the antibodies were applied. The tissue sections were incubated for 2 h in mouse anti-SSEA-4 FITC-conjugated (diluted 1:200) or mouse anti-SOX2 PE-conjugated (diluted 1:100) antibodies (both BD Biosciences). After rinsing the tissue sections several times with PBS, the slides were mounted with Vectashield mounting medium with DAPI (Vector Laboratories) and observed under fluorescent microscope. After DAPI staining the cell nuclei were stained blue. The SSEA-4-positive cells expressed surface green fluorescence and SOX2-positive cells expressed nuclear red fluorescence. We focused on SSEA-4 and SOX2-positivity matched with blue DAPI staining of nuclei.

## Results

### Ovarian surface epithelium

**Fig. 1:**
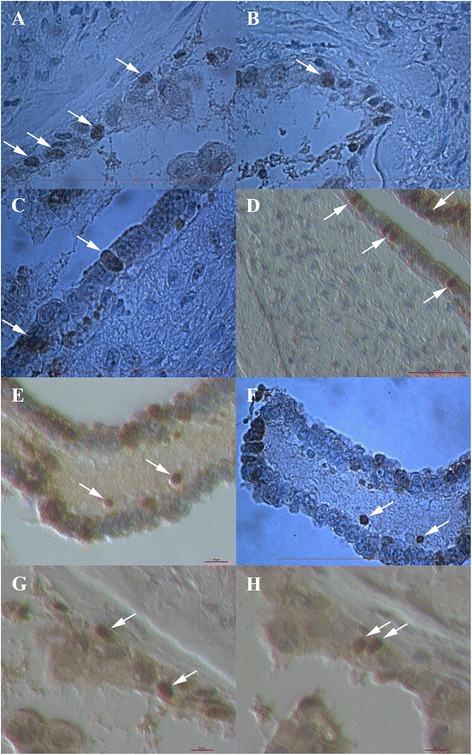
NANOG-positive cells (arrows) in the ovarian surface epithelium. They appeared among epithelial cells (**a**-**d**, **g**, **h**) without any special order, or just below them (**e** and **f**) in women with borderline ovarian cancer and high-grade serous ovarian carcinoma. (Light microscope: **a**-**c**, **f**, magnification 1000x; inverted microscope: **d**, **e**, **g**, **h**, magnifications 100x or 200x). *Legend*: brown-NANOG-positivity, blue-nuclei after HE staining. *Red Bar*: 100 μm for **a**-**c**, **f**, 50 μm for **d** and 10 μm for **e**, **g**, **h**

As expected, small round cells were observed in ovarian surface epithelium after HE staining and some of them also expressed NANOG-positivity (brown staining) in both ovarian tissue sections of women with borderline ovarian cancer and high-grade serous ovarian carcinoma (Fig. 1). The NANOG-positive cells were of sizes up to 5 μm and were located among epithelial cells without any special order (Fig. 1a-d, g, h) or just below them (Fig. 1e and f). The entire cells were positively stained for NANOG due to large nuclei filling almost the whole cell volumes; in some cells it was clear that they had almost no cytoplasm around the positively stained nuclei (e.g., Fig. 1b and c). The NANOG-positive staining matched with blue HE staining of nuclei.

### Small round cells in non-defined “chambers”

**Fig. 2:**
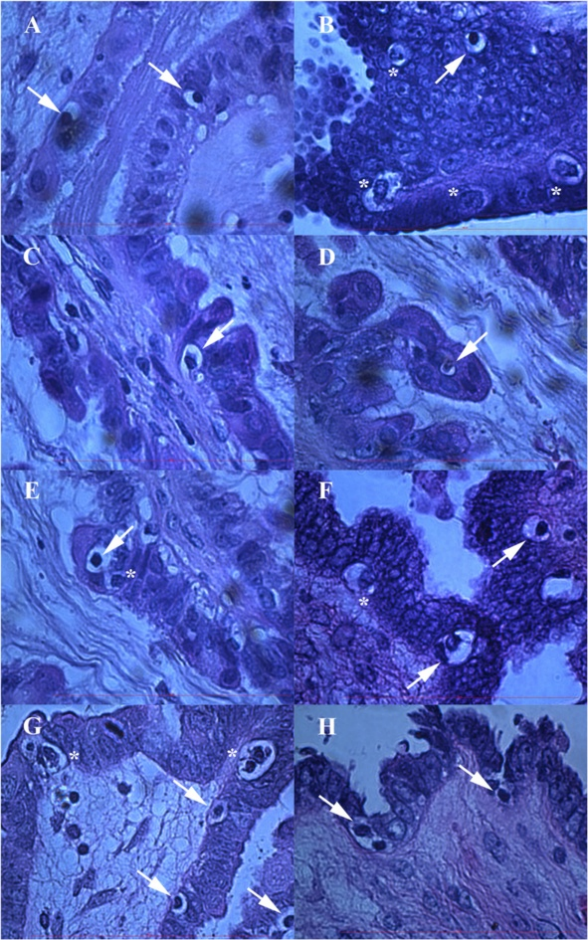
Special “chambers” containing small, round cells (arrows) or groups/clusters (asterisks) of these cells. The diameter of these small, round cells was up to 5 μm with nuclei filling almost the whole cell volumes. They were located among epithelial cells or just below them in ovarian sections of women with borderline ovarian cancer after hematoxylin-eosin (HE) staining (**a**-**h**). In the vicinity of these “chambers” the ovarian surface epithelium was drastically changed with several layers of proliferated epithelial cells, epithelial cells of atypical shapes (e.g., extended) and formation of papillae. (Light microscope, magnification 1000x). *Red Bar*: 100 μm

**Fig. 3:**
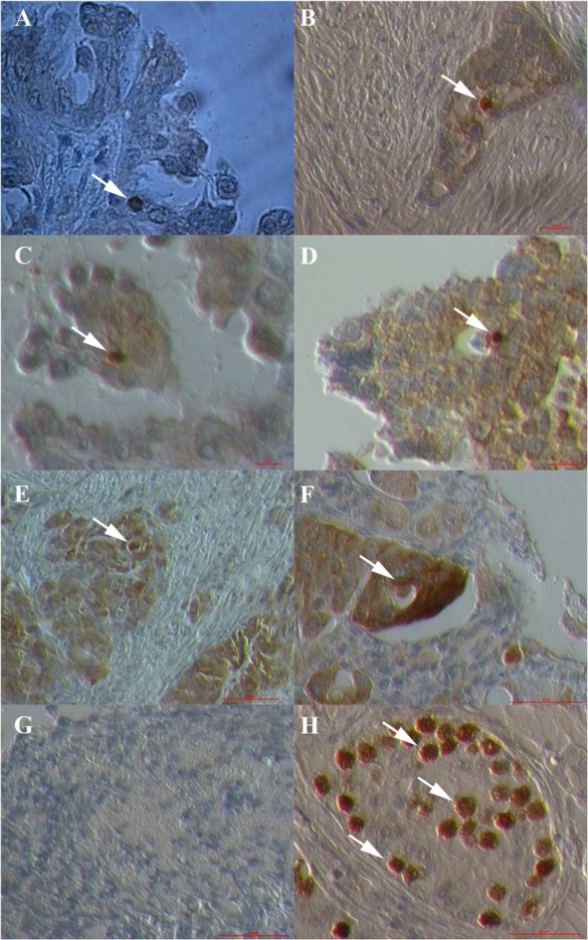
Small NANOG-positive cells (arrows) with diameters of up to 5 μm in “chambers”. These “chambers” containing small, round, NANOG-positive cells were located among epithelial cells in ovarian tissue sections of women with high-grade serous ovarian carcinoma (**a**-**f**). The surrounding epithelial cells also became NANOG-positive in some places (**d**-**f**), as possibly influenced by small cells in those “chambers”. Controls were provided: negative control (**g**) and positive control-testicular embryonal carcinoma with strongly stained cells in seminiferous tubules (**h**) (Light microscope: **a**, magnification 1000x; inverted microscope: **b**-**h**, magnifications 40x, 100x and 200x). *Legend*: brown-NANOG-positivity, blue-nuclei after HE staining. *Red Bar*: 10 μm for **b**-**d**, 50 μm for **f**, **h** and 100 μm for **a**, **e**, **g**

**Fig. 4:**
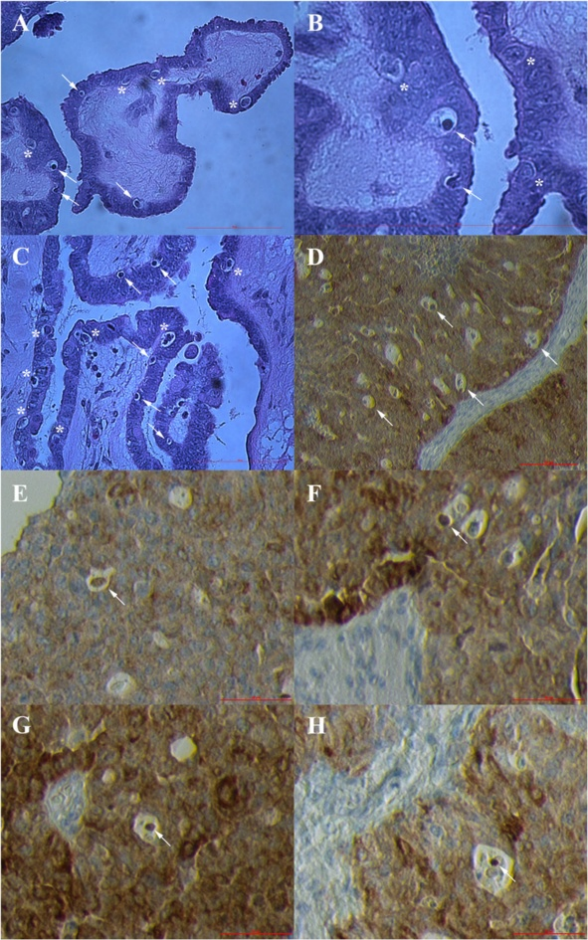
Highly abundant “chambers” containing small, round cells (arrows) or clusters of cells (asterisks) in ovarian tissue. They were present in borderline ovarian cancer (**a**-**c**) and high-grade serous ovarian carcinoma (**d**-**h**) tissue. In a proportion of “chambers” the small, round cells were NANOG-positive and were stained brown. (Light microscope: **a**-**c**, magnifications 200x or 1000X; inverted microscope: **d**-**h**, magnifications 40x and 100x). *Legend*: brown-NANOG-positivity, blue-nuclei after HE staining. *Red Bar*: 100 μm for **a**-**d** and 50 μm for **e**-**h**

**Fig. 5:**
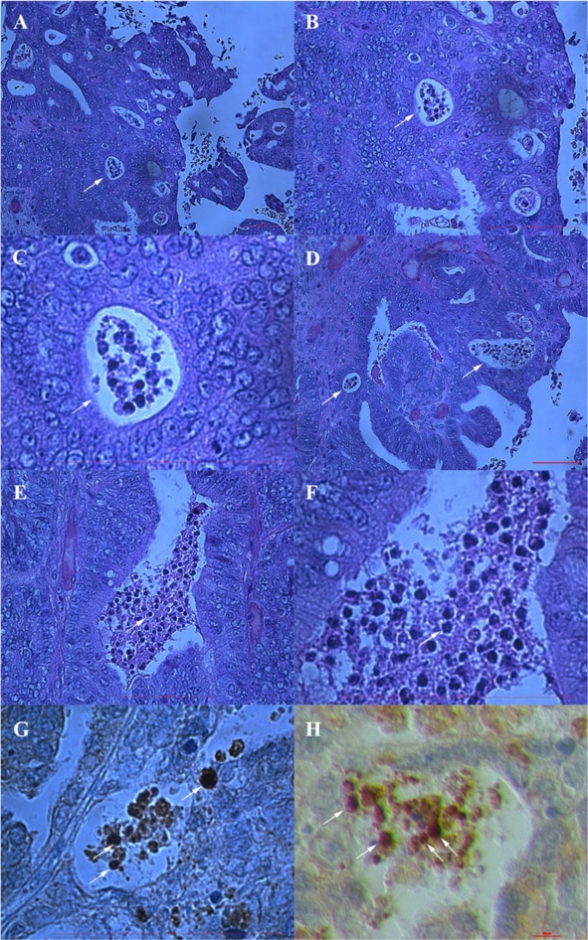
Ovarian tissue sections with “chambers” of different shapes containing groups of small, round cells (arrows). These small cells had diameters of up to 5 μm. Their nuclei filled almost the whole cell volumes in two patients with high-grade serous ovarian carcinoma after HE staining (**a**-**f**). A proportion of these cells were NANOG-positive (**g** and **h**). “Chambers” were surrounded by epithelial/cancer cells. Figures **a**, **b**, **c**, **e** and **f** show the same structures at different magnifications to get better insight. (Light microscope: **a**-**g**, magnifications 100x, 200x and 1000x; inverted microscope: **h**, magnification 200x). *Legend*: brown-NANOG-positivity, blue-nuclei after HE staining. *Red Bars*: 10 μm for h and 100 μm for **a**-**g**

In addition to NANOG-positive cells among epithelial cells of ovarian surface epithelium, there were also small single cells of the same sizes or groups of cells, which were NANOG-positive and were located in special, non-defined “chambers” of unknown origin. These “chambers” seem to be special structures with empty space, containing single or groups of small cells with diameters of up to 5 μm, and were arranged tightly among other types of cells in both borderline ovarian cancer and high-grade serous ovarian carcinoma (Figs. 2, 3, 4 and 5 and Additional file 1: Figure S1). In some “chambers” these small cells seem to divide and form cluster-like structures. They appeared in several places of completely non-damaged ovarian tissue and were not a matter of tissue degradation. The “chambers” with small cells were clearly seen after HE staining, as shown in Fig. 2 and were present in ovarian sections of all women included in this study who suffered from both borderline ovarian cancer and serous ovarian carcinoma. In spite of this, our impression was that these “chambers” were more abundant in the tissue of borderline ovarian cancer than in serous ovarian carcinoma, while present in both types of tissue. It was impossible to count these “chambers” because they were quite abundant. Cells in these “chambers” were small, round and with nuclei that filled almost the whole cell volumes. The “chambers” were mostly located in the ovarian surface epithelium layer among epithelial cells, just below it or inside the “islands” of epithelial cells inside the ovarian tissue (Figs. 2 and 3, Additional file 1: Figure S1). In the vicinity of these “chambers” the ovarian surface epithelium was morphologically changed: epithelial cells had proliferated, appearing in more layers, and the typical shape of epithelial cells was drastically changed (e.g., extended cells), as shown in Fig. 2 and Additional file 1: Figure S1. In two patients with high-grade serous ovarian carcinoma these “chambers” were spread over all ovarian tissue sections, as can be seen in Fig. 4. Moreover, two patients with serous ovarian carcinoma showed big, specific “chambers” appearing in the tissue, which contained several small, separated cells with diameters of up to 5 μm and nuclei filling almost the whole cell volumes (see Fig. 5). These “chambers” were not blood vessels because there were no erythrocytes or other typical blood cells in them, and they were not surrounded by muscular but epithelial/cancer cells. Interestingly, comparable “chambers” including oocyte progenitor cells were found in fetal ovaries yet were not present in the ovaries of healthy women without cancer (Additional file 2: Figure S2).

A proportion of small round cells in such “chambers” was positively stained for NANOG (Figs. 3, 4 and 5). In NANOG-positive cells the whole cells were stained brown, thus indicating that the nuclei filled almost the whole cell volumes. Beside this, in the regions with “chambers”, including the NANOG-positive cells, the surrounding epithelial cells were also more or less positively stained for NANOG (Figs. 3d-f and 4f-h), while more distant cells were not.

### Tumor-like structures in “chambers”

**Fig. 6:**
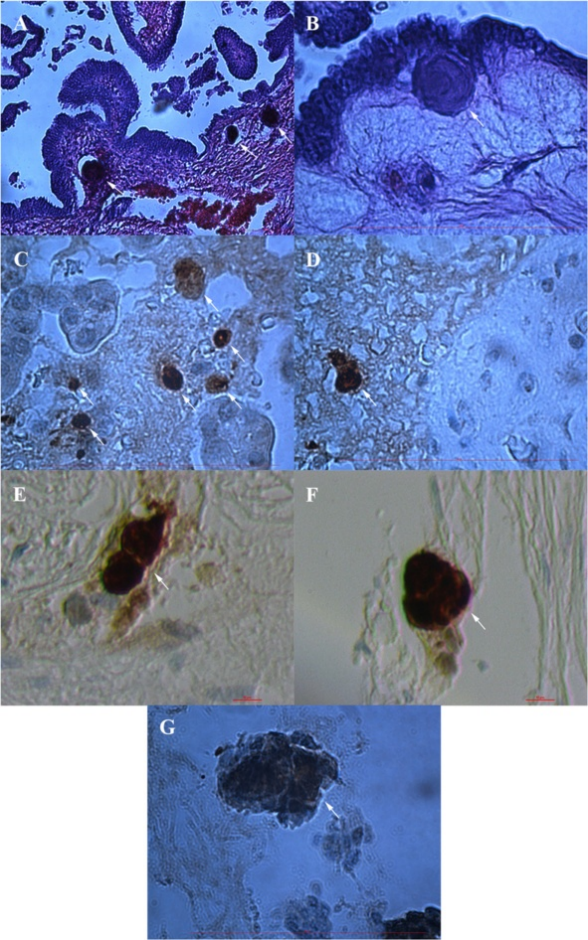
Tumor-like structures (arrows) developed in “chambers” present in ovarian tissue (**a**-**g**). They were present in ovaries of women with borderline ovarian cancer and high-grade serous ovarian carcinoma. The tumor-like structures were strongly NANOG-positive (**c**-**g**). A small proportion of tumor-like structures in borderline ovarian cancer was mineralized and with no visible nuclei (**b**). Some tumor-like structures were composed of small cells with clearly visible nuclei (**g**). (Light microscope: **a**-**d**, **g**, magnification 100x and 1000x; inverted microscope: **e**-**f**, magnification 200x). *Legend*: brown-NANOG-positivity, blue-nuclei after HE staining. *Red Bars*: 100 μm for **a**-**d**, **g** and 10 μm for **e**, **f**

In a relatively small proportion of the “chambers” some structures with diameters of up to 50 μm and resembling tumors were observed after HE staining (Fig. 6a and b). They were “closed” in growing “chambers”, although the tissue in their vicinity was mostly damaged and blood vessels were found to be close to them. Similar structures were found in ovaries from women with both borderline ovarian cancer and serous ovarian cancer. These tumor-like structures were clearly seen after HE staining of ovarian tissue (Fig. 6a and b), and very similar structures were positively stained for NANOG, as can be seen in Fig. 6c-g. A small proportion of tumor-like structures in the same “chambers” were mineralized (Fig. 6b) but in borderline ovarian cancer only; it was no longer possible to see the nuclei in these structures. On the other hand, it was clearly visible that some of tumor-like structures were composed of small cells (Fig. 6g), which were NANOG-positive, especially in the ovarian tissue of women with serous ovarian carcinoma.

### Primitive oocyte-like cells in “chambers”

**Fig. 7:**
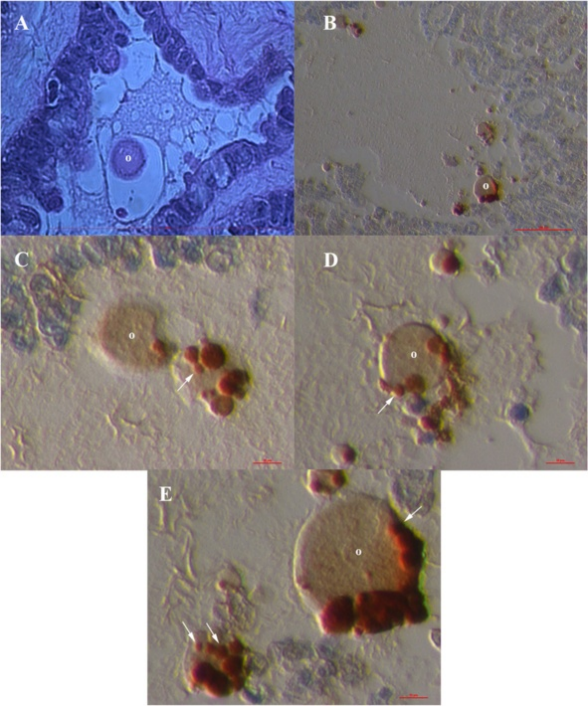
Primitive oocyte-like cells in “chambers” present in ovarian tissue. They were present in borderline ovarian cancer (**a**) and high-grade serous ovarian carcinoma tissue (**b**-**e**). The primitive oocyte-like cells were slightly NANOG-positive (**b**-**e**) and small, round, NANOG-positive cells (arrows) were attached to them. The oocyte-like cells appeared from these small cells inside the ovarian tumor. The structure of cytoplasm in these cells was quite comparable to human oocytes. Figures **b**-**e** show the same structures at different magnifications to get better insight. (Light microscope: **a**, magnification 1000x; inverted microscope: **b**-**e**, magnifications 200x). *Legend*: O-oocyte-like cell. *Legend*: brown-NANOG-positivity, blue-nuclei after HE staining. *Red Bars*: 100 μm for (**a**), 50 μm for (**b**) and 10 μm for (**c**-**e**)

Interestingly, primitive oocyte-like cells were found in some similar but rare “chambers” (Fig. 7). These cells were round, with diameters of up to 30 μm, and some of them expressed tiny zona pellucida-like structures (Fig. 7a). The primitive oocyte-like cells were present in these non-defined “chambers” and not in follicles (Additional file 2: Figure S2). These primitive oocyte-like cells were observed in the ovarian tissue of women with borderline ovarian cancer after HE staining (Fig. 7a) and also in women with serous ovarian carcinoma (Fig. 7b-e). The primitive oocyte-like cells, which can be seen in Fig. 7b-e were developed in the ovarian tissue of one patient with serous ovarian carcinoma and were positively stained for NANOG. The structure of their cytoplasm was quite comparable to human oocytes. Actually, the primitive oocyte-like cells appeared inside the ovarian tumor of high-grade serous carcinoma. Moreover, these primitive oocyte-like cells seem to develop from small round cells with diameters of up to 5 μm, which were strongly positive for NANOG, and were attached to them, as can be seen from Fig. 7b-e. These small, NANOG-positive cells were growing and reached different sizes up to 30 μm.

### SSEA-4 and SOX2-positive cells in ovarian sections

**Fig. 8:**
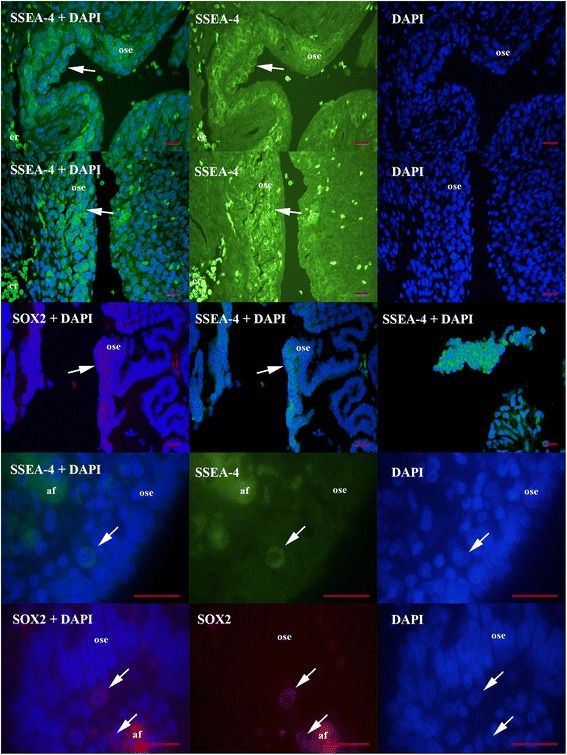
Small putative stem cells (arrows) expressing SSEA-4 and SOX2-positivity after immunohistochemistry and blue nuclei after DAPI staining of ovarian sections. Small SSEA-4 and SOX2-positive cells were proliferated in ovarian surface epithelium or were found in typical “chambers” with atrophic and autofluorescent (af) tissue nearby. *Legend*: green-SSEA-4-positivity, red-SOX2-positivity, ose-ovarian surface epithelium, af-autofluorescence, er-erythrocytes (without nuclei). *Red bars*: 10 μm

Similar populations of SSEA-4 and SOX2-positive cells were found in ovarian surface epithelium and typical “chambers”, as can be seen in Fig. 8. The small SSEA-4 and SOX2-positive cells with diameters of up to 5 μm were proliferated in the layer of ovarian surface epithelium. Single small SSEA-4 and SOX2-positive cells were present in “chambers” and atrophic (autofluorescent) ovarian tissue was found in their vicinity. This observation further confirmed the above findings on NANOG expression.

## Discussion

The aim of our study is not to provide a new diagnostic tool for ovarian cancer but to identify in situ the small putative stem cells, which previously had been discovered in human ovarian cell cultures in vitro. They were found in the ovarian surface epithelium. Furthermore, our observation showed that in ovarian tissue sections from patients with borderline ovarian cancer and high-grade serous ovarian carcinoma there are non-defined “chambers” of unknown origin. These “chambers” are interesting because they contain small round cells with diameters of up to 5 μm or groups/clusters of these cells, which were positively stained for pluripotency-related NANOG. Moreover, in some of these “chambers” tumor-like structures and primitive oocyte-like cells were observed. Our data indicate that these “chambers” may include putative stem cells, which may be involved in tumor formation and oogenesis.

The results of this study in situ may confirm the previous finding that in adult human ovaries [19–21, 24, 25] and ovaries of some other mammalian species [25–27] such as rabbit, sheep, marmoset monkey, pig and mouse there is a population of small stem cells with diameters of about 2 to 4 μm, which express a specific pattern of pluripotency, including NANOG [24], and resemble VSELs discovered in adult human bone marrow [28, 29], peripheral blood [30] and cord blood [31] by the Ratajczak research group. These small stem cells are mostly located in the ovarian surface epithelium of adult ovaries [19–21, 23, 25]. Also in this study small, NANOG-positive cells were indeed found in the layer of ovarian surface epithelium among epithelial cells or just below them. Other than in “healthy” ovaries, similar small, NANOG-positive cells were present in special “chambers” of unknown origin in the ovaries of women with borderline ovarian cancer or high-grade serous ovarian carcinoma. The small round cells possessed two important features of stem cells: nuclei filling almost the whole cell volumes and strong nuclear staining for pluripotency-related marker NANOG. Similar populations of SOX2 and SSEA-4-positive cells were found in ovarian sections of women with high-grade serous ovarian carcinoma and their expression nicely matched with NANOG expression.

We were searching the literature for a description of such “chambers” but were able to find only data on psammoma bodies [70–73], which are described as round collections of calcium with a laminar appearance and which are usually associated with the papillary histomorphology of different cancers, including serous ovarian carcinoma, and are thought to arise from the infarction and calcification of papillae tips and calcification of intralymphatic tumor thrombi. The psammoma bodies can also appear in chamber-like arrangements, but our data clearly show that cancerous ovarian tissue “chambers” definitely do not contain calcium, instead, small, round cells or clusters of these cells are present. These small NANOG-positive cells might be proposed as lymphocytes (i.e., immunological infiltrates), but lymphocytes, in general, are of bigger sizes; small lymphocytes have diameters of 7 to 10 μm and large lymphocytes from 14 to 20 μm according to the Atlas of Microscopic Anatomy [74]. Moreover, the lymphocytes definitely do not express the pluripotency-related marker NANOG. The specific “chambers” that contain small NANOG-positive cells may further be proposed to be free spaces filled with cell debris in atrophic parts of ovarian tissue; however, the special “chambers” observed in this study also appeared in completely undamaged ovarian tissue and contain completely round cell-like structures with blue stained nuclei, which positively stained for NANOG. Upon such description, one may propose these “chambers” to be vacuolated cancer cells and autophagy, but this phenomenon looks completely different when observed in situ [75].

Moreover, tumor-like structures and even primitive oocyte-like cells, positive for NANOG, were found in similar “chambers”, indicating that they might develop from small putative stem cells expressing NANOG, within these “chambers”. Our preliminary experimental data, published as a case report in this journal, showed that small cells in “chambers” in the ovaries of an aged woman with serous papillary adenocarcinoma have also expressed other markers of pluripotency and germinal lineage, which were proposed to be primitive germ cells either persisting from the fetal period of life or developed from putative stem cells [76]. Other tissue in which we were indeed able to observe similar “chambers” containing small cells was the fetal ovaries. In fetal ovaries, the tissue was expanded by comparable “chambers”, which contained oocyte progenitor cells. Based on this observation, some similarity between the morphology of cancerous ovaries and fetal ovaries can be suggested. An important question arose as to whether the early follicles, i.e., “chambers”, containing pluripotent germ cell progenitors, persisting from the fetal period of life, may be related to the manifestation of ovarian cancer. In our previous research, small stem cells have been observed in ovarian surface epithelium of “healthy” ovaries, but were not present inside such “chambers”.

At several places the epithelial cells in the vicinity of these “chambers” containing small putative stem cells were drastically changed; they had proliferated and appeared in more layers, and they expressed an atypical changed shape (e.g., formation of papillae). We suggest that small putative stem cells in cancerous ovaries, especially those in “chambers”, affect the surrounding epithelial cells and possibly trigger them to change by an unknown mechanism such as cell secretion, epithelial-mesenchymal transition [51–67] or something else. The experimental results from the literature indeed showed that cancer cells in epithelial ovarian cancers express a strong cytoplasmic expression of pluripotency-related NANOG [69].

The observation of NANOG-positive primitive oocyte-like cells in “chambers” of cancerous ovaries, which very possibly developed from small putative stem cells, strongly expressing NANOG and are attached to them, confirms the previous observation on small putative stem cells in adult human ovaries that may develop into primitive oocyte-like cells in vitro, for example in the presence of follicular fluid rich on substances important for oocyte growth and maturation in humans [19, 20, 22, 25, 37] and in some other mammalian species such as mouse [26], pig [27], and marmoset monkey [77]. The development of stem cells into oocyte-like cells in vitro represents an important confirmation of the pluripotent character of stem cells, as experienced in embryonic stem cells (ESCs) [78] and human induced pluripotent stem cells (hiPSCs) [79].

## Conclusions

The small putative stem cells with diameters of up to 5 μm, expressing pluripotency-related markers NANOG, SOX2 and SSEA-4 which were identified in the ovarian sections of the women suffering from borderline ovarian cancer or high-grade serous ovarian carcinoma included into this study, were present in the ovarian surface epithelium and in special, non-defined “chambers”. Our observation indicates that they may be involved in tumorigenesis and oogenesis. This observation is consistent with the idea of very small embryonic-like stem cells (VSELs), which have already been discovered in other adult human tissues and organs, such as bone marrow, and are suggested to originate from embryonic epiblast, relate to primordial germ cells (PGCs) that persist from the embryonic period of life and are involved in the regeneration of damaged tissues and organs. On the other hand, these small stem cells may form tumors in inappropriate conditions in the body [32–36]. Our results indicate that further research of small putative stem cells and “chambers” in situ is needed through the application of other markers, related to stem cells, pluripotency, germinal lineage, and cancer in order to maybe better understand the manifestation of ovarian cancer in the future.

## Additional files

Additional file 1: Figure S1. “Chambers” (arrows) containing small cells in ovarian surface epithelium of women with borderline ovarian cancer. Epithelial cells in the vicinity of “chambers” were drastically changed in terms of shape and proliferation (a-f), as observed after HE staining. (Inverted microscope: a-e, magnification 200x; light microscope: f, magnification 1000x). *Legend*: blue-nuclei, e-epithelial cells. *Red Bars*: 10 μm for a-e and 100 μm for f. (JPG 1446 kb) Additional file 2: Figure S2. Sections of ovaries in borderline ovarian cancer, fetal ovary, and women of reproductive age. In borderline ovarian cancer (a) and fetal ovary (b) comparable “chambers” (arrows) containing small round cells have been observed. In “chambers” of fetal ovaries the oocyte progenitor cells are present. No comparable “chambers” have been observed in ovaries of women of reproductive age: fertile women (c) with follicles in ovarian cortex, and women with premature ovarian failure (d) without follicles in the ovarian cortex. In cancerous ovaries early follicles containing primitive oocytes were still found in “normal” ovarian cortex tissue (e). (Light microscope: a-e, magnifications 100x, 200x and 1000X). *Legend*: F-follicle. *Red Bars*: 100 μm. (JPG 1981 kb)

## Abbreviations

HE: hematoxylin and eosin
MSCs: mesenchymal stem cells
NANOG: nanog homeobox
VSELs: very small embryonic-like stem cells
