# Supplementary figures and images for: Small putative NANOG, SOX2, and SSEA-4-positive stem cells resembling very small embryonic-like stem cells in sections of ovarian tissue in patients with ovarian cancer
Source: J Ovarian Res. 2016 Mar 3;9:12. doi: 10.1186/s13048-016-0221-3 (PMC4778328; doi:10.1186/s13048-016-0221-3)

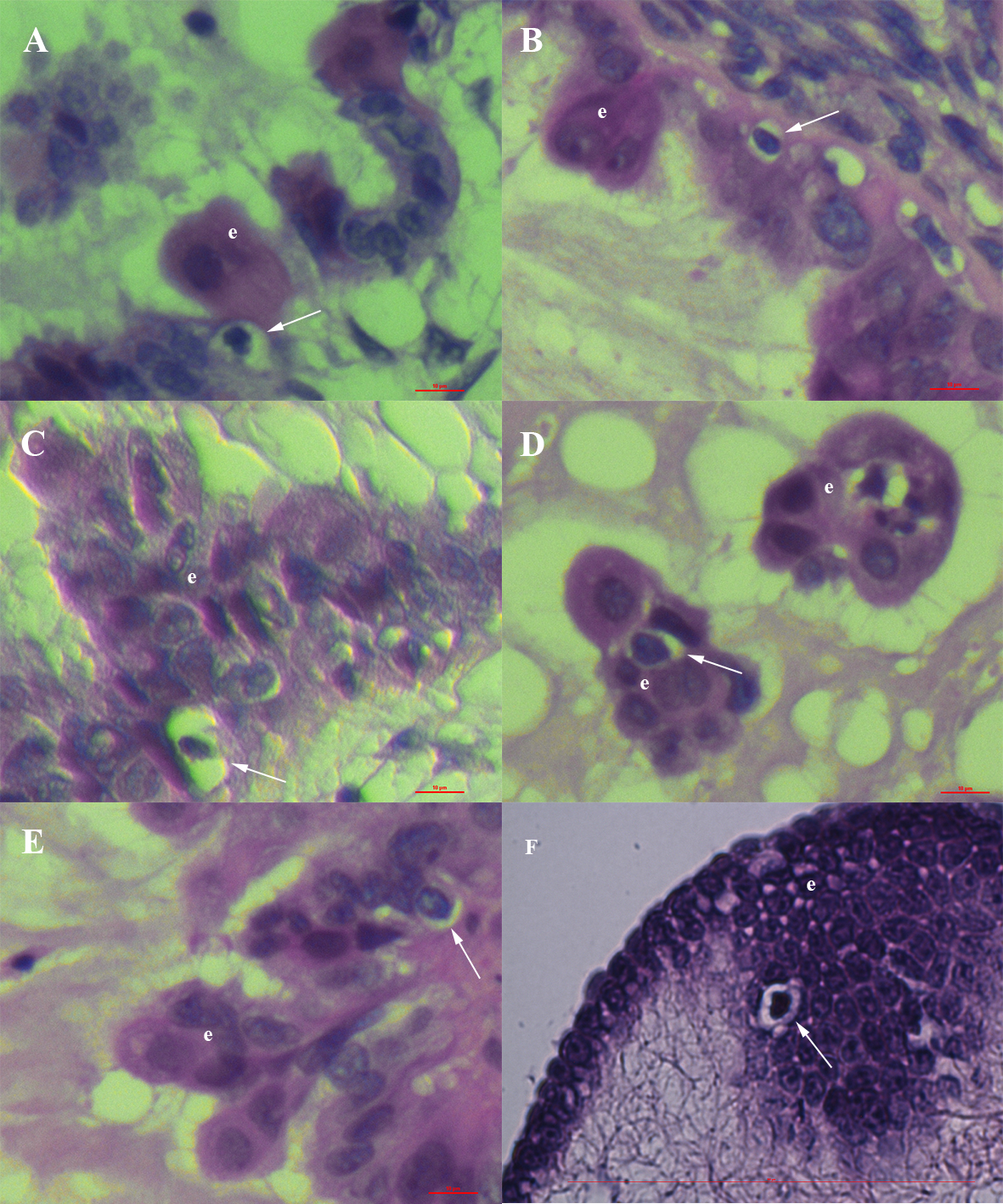

Supplement: Additional file 1: Figure S1. — “Chambers” (arrows) containing small cells in ovarian surface epithelium of women with borderline ovarian cancer. Epithelial cells in the vicinity of “chambers” were drastically changed in terms of shape and proliferation (a-f), as observed after HE staining. (Inverted microscope: a-e, magnification 200x; light microscope: f, magnification 1000x). Legend: blue-nuclei, e-epithelial cells. Red Bars: 10 μm for a-e and 100 μm for f. (JPG 1446 kb) [file 13048_2016_221_MOESM1_ESM.jpg]

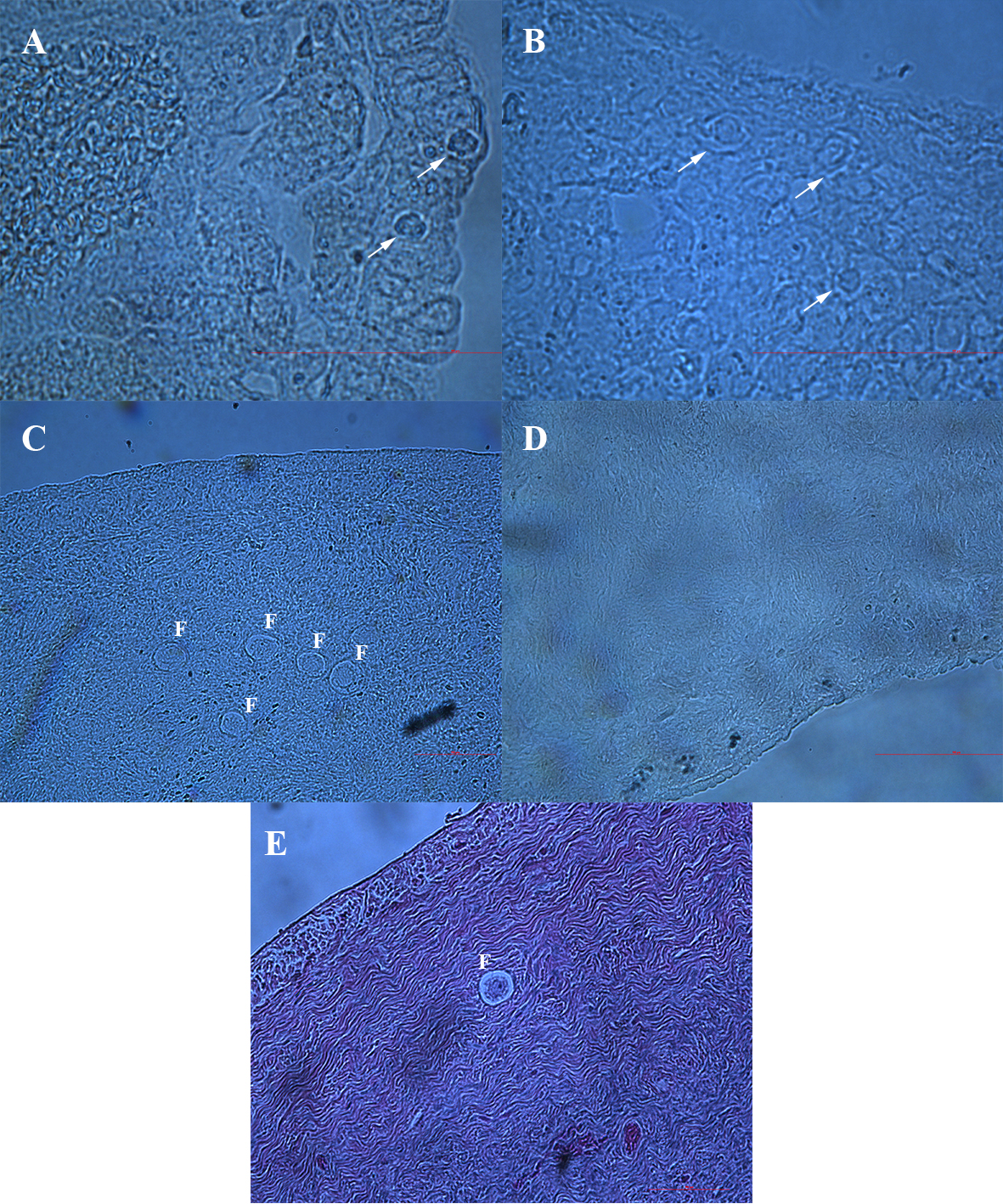

Supplement: Additional file 2: Figure S2. — Sections of ovaries in borderline ovarian cancer, fetal ovary, and women of reproductive age. In borderline ovarian cancer (a) and fetal ovary (b) comparable “chambers” (arrows) containing small round cells have been observed. In “chambers” of fetal ovaries the oocyte progenitor cells are present. No comparable “chambers” have been observed in ovaries of women of reproductive age: fertile women (c) with follicles in ovarian cortex, and women with premature ovarian failure (d) without follicles in the ovarian cortex. In cancerous ovaries early follicles containing primitive oocytes were still found in “normal” ovarian cortex tissue (e). (Light microscope: a-e, magnifications 100x, 200x and 1000X). Legend: F-follicle. Red Bars: 100 μm. (JPG 1981 kb) [file 13048_2016_221_MOESM2_ESM.jpg]
